# Prognostic role of platelet to lymphocyte ratio in esophageal cancer: A meta-analysis

**DOI:** 10.18632/oncotarget.22557

**Published:** 2017-11-20

**Authors:** Qing-Tao Zhao, Xiao-Peng Zhang, Hua Zhang, Guo-Chen Duan

**Affiliations:** ^1^ Department of Thoracic Surgery, Hebei General Hospital, Shijiazhuang 050051, Hebei, PR China

**Keywords:** platelet to lymphocyte ratio, esophageal cancer, prognosis, meta-analysis

## Abstract

**Purpose:**

The prognostic role of inflammation index like platelet to lymphocyte ratio (PLR) in esophageal cancer remains controversial. We evaluated the prognostic significance of PLR in esophageal cancer patients.

**Methods:**

We searched databases to identify relevant literatures. Pooled hazard ratios (HRs) and 95% confidence intervals (CIs) were calculated. A meta-analysis was performed to evaluate the prognostic value of PLR in patients with esophageal cancer.

**Results:**

A total of 6,699 patients from 16 studies (17 cohorts) were finally enrolled in the meta-analysis. The results demonstrate that the elevated PLR predicted poorer overall survival (OS) (HR: 1.389, 95% CI: 1.161-1.663) and disease-free survival (DFS) (HR: 1.404, 95% CI: 1.169-1.687) and cancer-specific survival (CSS) (HR: 1.686, 95% CI: 1.146-2.480) in patients with esophageal cancer. Subgroup analysis revealed that the elevated PLR was also associated with poor OS in esophageal cancer treated by surgery (HR: 1.492, 95%CI: 1.149-1.938, P<0.05) and mixed treatment (HR: 1.222, 95%CI: 1.009-1.479, P<0.05). In addition, PLR Cut-off value≤160 (HR: 1.484, 95%CI: 1.088-2.024, P<0.05) and PLR Cut-off value>160 (HR: 1.391, 95%CI: 1.161-1.666, P<0.05).

**Conclusion:**

This meta-analysis result suggested that PLR might be a significant predicative biomarker of poor prognosis for esophageal cancer patients.

## INTRODUCTION

Esophageal cancer is one of the most common cancers in the worldwide [1]. Despite the research on the treatment of esophageal cancer and the use of increasingly advanced technology in its treatment, the overall survival (OS) and progression-free survival (PFS) are still poor due to the high rate of recurrence and rapid progression [2, 3]. Therefore, there is an urgent need for us to identify better prognostic biomarkers, especially serum predictive biomarkers, for prognosis in patients with esophageal cancer.

Recently, more and more evidence showed that a systemic inflammatory response could play an important role in the prognosis of various cancers [4-7]. Various inflammatory factors, such as C-reactive protein (CRP), neutrophil to lymphocyte ratio(NLR), and platelet to lymphocyte ratio (PLR), have been investigated in various types of cancers [8, 9]. PLR, calculated as platelet counts divided by lymphocyte counts, has been associated with worse survival for a variety of cancers including the lung cancer [10], colorectal cancer [11], gastric cancer [12] and so on. Recent studies demonstrated a potential prognostic role of PLR in esophageal cancer patients [13-16]. However, due to the inconsistent results, the current opinion on the prognostic role of PLR in esophageal cancer remains controversial [13, 14, 16]. We therefore conducted a meta-analysis to comprehensively and systematically understand the prognostic value of PLR in esophageal cancer. In this study, we aimed at assessing the prognostic significance of high PLR for survival in patients with esophageal cancer.

## RESULTS

### Study characteristics

**Figure 1:**
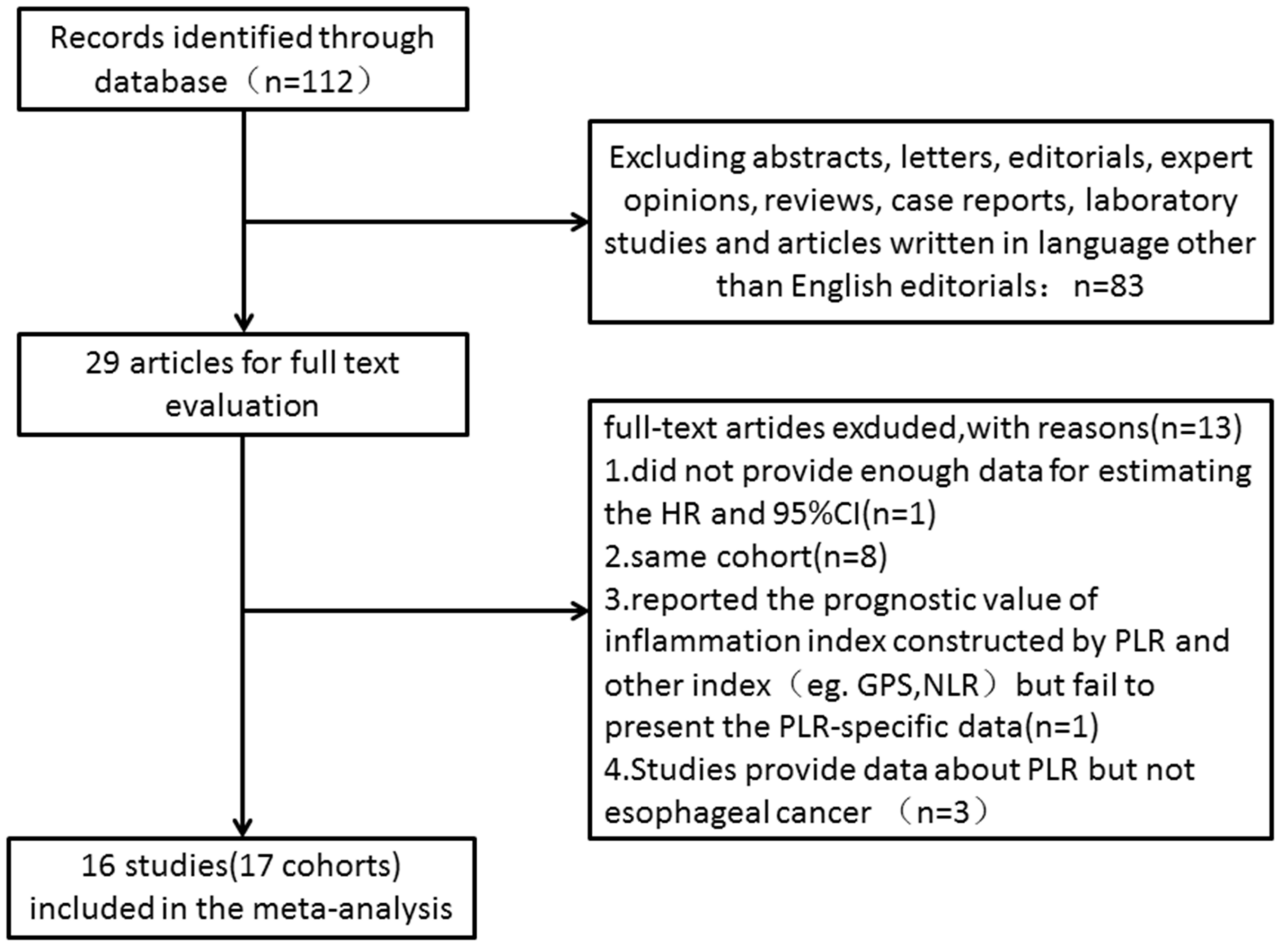
Flow chart of the included studies

Our database search retrieved a total of 112 articles. We eliminated 83 articles for various reasons based on the title and abstract, leaving 29 studies to scrutinize with a full text review. Of the 29 studies, three were reported by the same study center and the patients were overlapping or partly overlapping in the studies [15, 17, 18]. To avoid duplicate counting, only one study with more complete data was selected [15]. Therefore, 16 studies (17 cohorts) with a total of 6699 patients published between 2011 and 2017 were finally enrolled in our meta-analysis [13-16, 19-30]. The processes of study selection were summarized in the flow diagram (Figure 1). The detailed characteristics of all included studies are summarized in Table 1. All 17 were retrospective observational cohort studies. The 16 included studies evaluated a total of 6699 patients, including 6303 with squamous cell carcinoma, 353 with adenocarcinoma, 43 with small cell carcinoma. We evaluated studies from 4 different countries, including 10 studies from China, 2 from Japan, 2 from the United States and 2 from the United Kingdom.

**Table 1:** Main characteristics of all the studies included in the meta-analysis

Study cohort	Year	Study region	No. (M/F)	Follow-up (months) (median and range)	Treatment	Age (years) (median and range)	cut-off	Outcome	Stage	Type	HR	NOS score
Hu D1	2017	China	1822(1822/0)	38.2(0.5-180)	Surg	55.98±9.81	NR	mortality	I-III	SCC	R(U/M)	6
Hu D2	2017	China	574(0/574)	38.2(0.5-180)	Surg	57.93±9.41	NR	mortality	I-III	SCC	R(U/M)	6
McLaren PJ	2017	USA	60(48/12)	NR	Surg/+Neo CMRT	66	NR	OS	I-IV	ADC/SCC	R(M)	4
Chen PC	2016	China	323(281/42)	NR	Surg	59.1± 7.9	150	CSS	I-III	SCC	R(U/M)	5
Geng Y	2016	China	916(696/220)	39(3-146)	Surg	60(37-84)	120	OS	I-III	SCC	R(U/M)	6
Hirahara N	2016	Japan	147(132/15)	42(3-111)	Surg	<70, n=46; ≥70, n=101	147	OS/CSS	I-IIIc	SCC	R(U)	6
Toyokawa T	2016	Japan	185(152/33)	81.5(IQR 45.8–112.3)	Surg/±Neo CMRT/Neo CMT/Neo RT	<65, n=95; ≥60, n=90	193	OS/RFS	I-IV	SCC	R(M)	6
Zhang F	2016	China	468(376/92)	49.1±32.6(3.2-114.5)	Surg	60(36-81)	212	OS/DFS	I-III	SCC	R(U/M)	6
Han LH	2015	China	218(177/41)	38.6(3-71)	Surg	60.5(32-84)	244	OS/DFS	I-III	SCC	R(U/M)	6
Hyder J	2015	USA	83(72/11)	29.3	Surg/+Neo CMRT	59(26-82)	429.7	PFS	II-IV	ADC/SCC	R(U)	6
Messager M	2015	UK	153(128/25)	31.8(4-131)	Surg/±Adj CMT	64.9(39.9–81.6)	192	OS/DFS	I-III	ADC	R(U/M)	6
Xu XL	2015	China	468(416/52)	49.9(10.9–88.0)	Surg/±Adj CMT/±Adj CMRT	58	147	OS	I-IIIc	SCC	R(U)	6
Feng JF	2014	China	483(411/72)	NR	Surg	59.1±8.0(34-80)	150	OS	I-III	SCC	R(U/M)	5
Xie X	2014	China	317(244/73)	46(36-62)	Surg/±Adj CMT	58.1(34-76)	103	CSS	I-III	SCC	R(M)	6
Yuan D	2014	China	327(282/45)	24.7(2–39)	Surg/±Neo CMT/±Adj CMT	63.1±9.7(39-77)	300	OS/DFS	I-III	SCC	R(U)	6
Feng JF	2013	China	43(30/13)	NR	Surg/±Adj CMRT	58.7±7.8(45-74)	150	OS	I-III	Small cell	R(U)	4
Dutta S	2011	UK	112(85/27)	55	Surg/±Neo CMRT/±Adj CMRT	<65, n=68; ≥65, n=44	150	CSS	I-IV	ADC/SCC	R(U)	6

ADC adenocarcinoma, Adj adjuvant therapy, CMRT chemoradiotherapy, CMT chemotherapy, CSS cancer-specific survival, DFS disease-free survival, HR hazard ratio, Neo neoadjuvant, NOS Newcastle–Ottawa Quality Assessment Scale, NR not reported, OS overall survival, PFS progression-free survival, RFS relapse-free survival, SCC squamous cell carcinoma, Surg curative surgery, R obtained by reporting in text, M means the HR come from multivariate analysis, U means the HR comes from univariate analysis.

HRs and 95% CIs were reported directly in 16 studies, 11 of which calculated HRs by the multivariate analysis and 13 via univariable analysis. 9 of these cohorts had ≥300 patients and 8 cohorts enrolled <300 patients. The cut-off values applied in the studies were not consistent ranging from 103 to 429.7. 6 cohorts used a PLR greater than 160, while 8 cohorts had a PLR cutoff value of 160 or less. The characteristics of the included studies were shown in Table 1.

### PLR and OS in esophageal cancer

**Figure 2:**
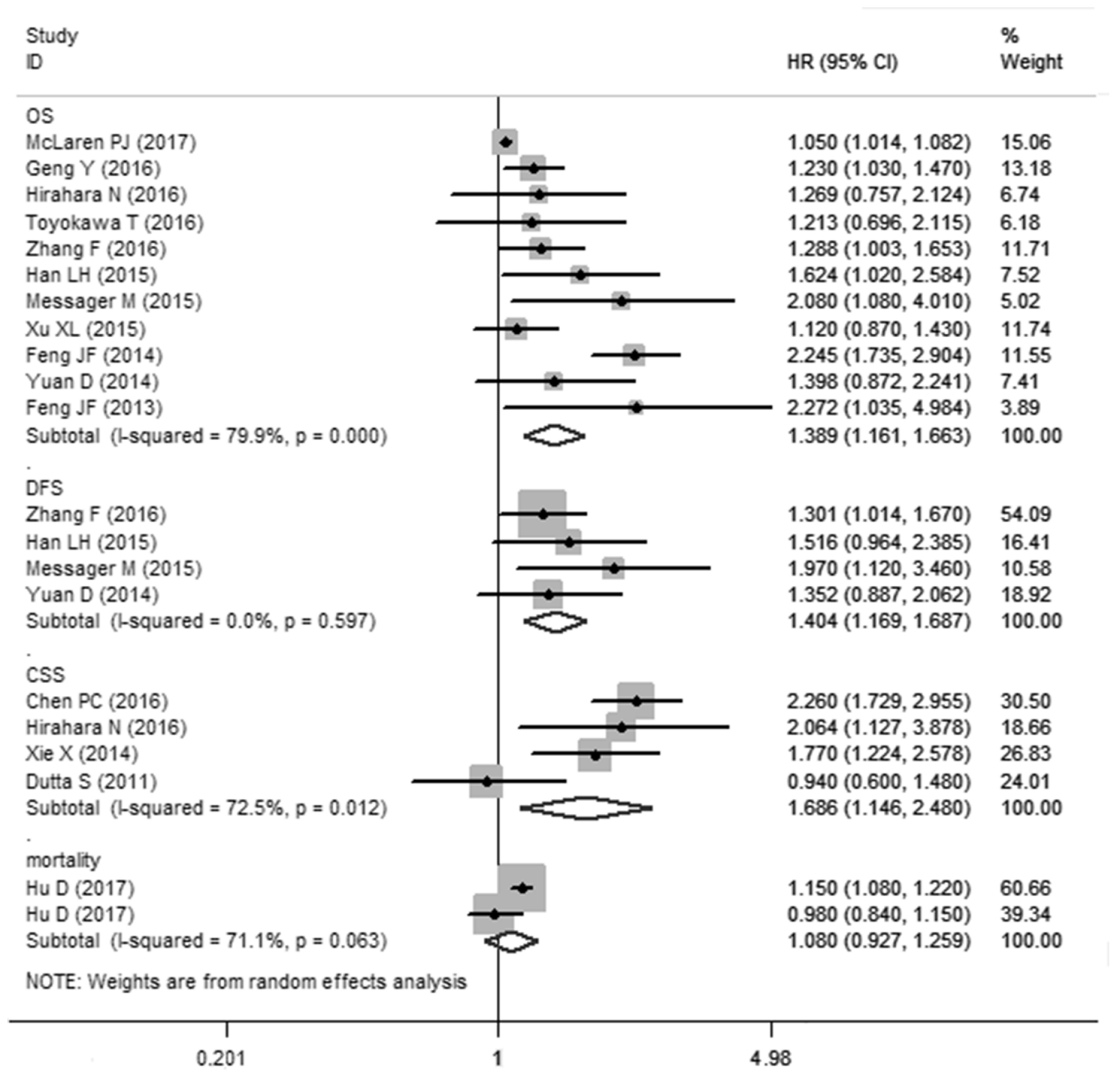
Meta-analysis of the association between PLR and OS/CCS/DFS/mortality of esophageal cancer

11 cohorts presented the relationship between PLR and OS in esophageal cancer. Though with significant heterogeneity (*I*^2^=79.9%, *Ph*<0.01), therefore, a random-effects model was applied. Our results revealed that elevated PLR were significantly correlated with worse OS (HR: 1.389, 95% CI: 1.161-1.663, *P*<0.001) (Figure 2). In a further investigation, subgroup analyses were performed (Table 2). 5 cohorts presented the information of PLR correlated with OS in esophageal cancer initially treated by surgery. we revealed the pooled HR was 1.492 (95%CI: 1.149-1.938, *P*<0.05) for patients treated by surgery and 1.222 (95%CI: 1.009-1.479, *P*<0.05) for patients treated by mixed treatment. In addition, subgroup analysis was performed by the sample size (≥300 and <300), cut-off value (cut-off value≤160 and cut-off value>160) and (Univariate analysis and Multivariate analysis).

**Table 2:** Summary of the meta-analysis results

Analysis	N	Random-effects model		Fixed-effects model		Heterogeneity		Meta-regression	
		HR(95%CI)	*P*	HR(95%CI)	*P*	*I*^2^	*Ph*	adj R^2^	*P*
OS	11	1.389(1.161,1.663)	0	1.079(1.046,1.112)	0	79.9%	0		
Subgroup 1: Surgery	5	1.492(1.149,1.938)	0.003	1.440 (1.279,1.621)	0	74.5%	0.003	10.82%	0.377
Mixed treatment	6	1.222(1.009,1.479)	0.04	1.056(1.023,1.090)	0.001	48.4%	0.085		
Subgroup 2: Cut-off value≤160	5	1.484(1.088,2.024)	0.013	1.396(1.237,1.576)	0	79.6%	0.001	63.83%	0.932
Cut-off value>160	5	1.391(1.161,1.666)	0	1.391(1.161,1.666)	0	0%	0.650		
Subgroup 3: sample size≥300	5	1.404(1.096,1.799)	0.007	1.368(1.226,1.526)	0	78.1%	0.001	70.20%	0.908
sample size<300	6	1.376(1.047,1.809)	0.022	1.057(1.023,1.091)	0.001	58%	0.036		
Subgroup 4:								63.33%	0.353
SCC		1.394(1.156,1.682)	0.001	1.370(1.236,1.518)	0	63.3%	0.008		
ADC		2.080(1.079,4.008)	-	2.080(1.079,4.008)	-	-	-		
Subgroup 5: Univariate analysis	9	1.477(1.219,1.789)	0	1.401(1.256,1.552)	0	63.4%	0.005	68.20%	0.215
Multivariate analysis	7	1.225(1.026,1.462)	0.024	1.064(1.032,1.098)	0	73.7%	0.002		
DFS	4	1.404(1.169,1.687)	0	1.404(1.169,1.687)	0	0%	0.597		
CSS	4	1.686(1.146,2.480)	0.008	1.814(1.505,2.186)	0	72.5%	0.012		
mortality	2	1.044(0.825,1.322)	0.718	1.133(1.070,1.200)	0	83.3%	0.014		

ADC: adenocarcinoma, CI: confidence interval, CSS cancer-specific survival, DFS disease-free survival, HR hazard ratio, Mixed treatment including adjuvant/neoadjuvant±chemotherapy/chemoradiotherapy/radiotherapy±surgery, N number of studies, OS overall survival, SCC squamous cell carcinoma, Ph p value of Q test for heterogeneity test.

### PLR and DFS and CSS in esophageal cancer

A fixed-effects model (*I*^2^=0%, *Ph*=0.597) was also used for studies evaluating DFS. Our results showed that elevated PLR predicted a worse outcome for DFS with the combined HR of 1.404 (95% CI: 1.169–1.687, *P*<0.001, Figure 2). Meta-analysis of these 4 studies showed that esophageal cancer patients with elevated PLR were associated with shorter CSS (HR obtained from Random-effects model:1.686, 95% CI: 1.146–2.480, *P*<0.01. Table 2) with obvious heterogeneity (*I*^2^=72.5%, *Ph*=0.012).

### Sensitivity analysis

**Figure 3:**
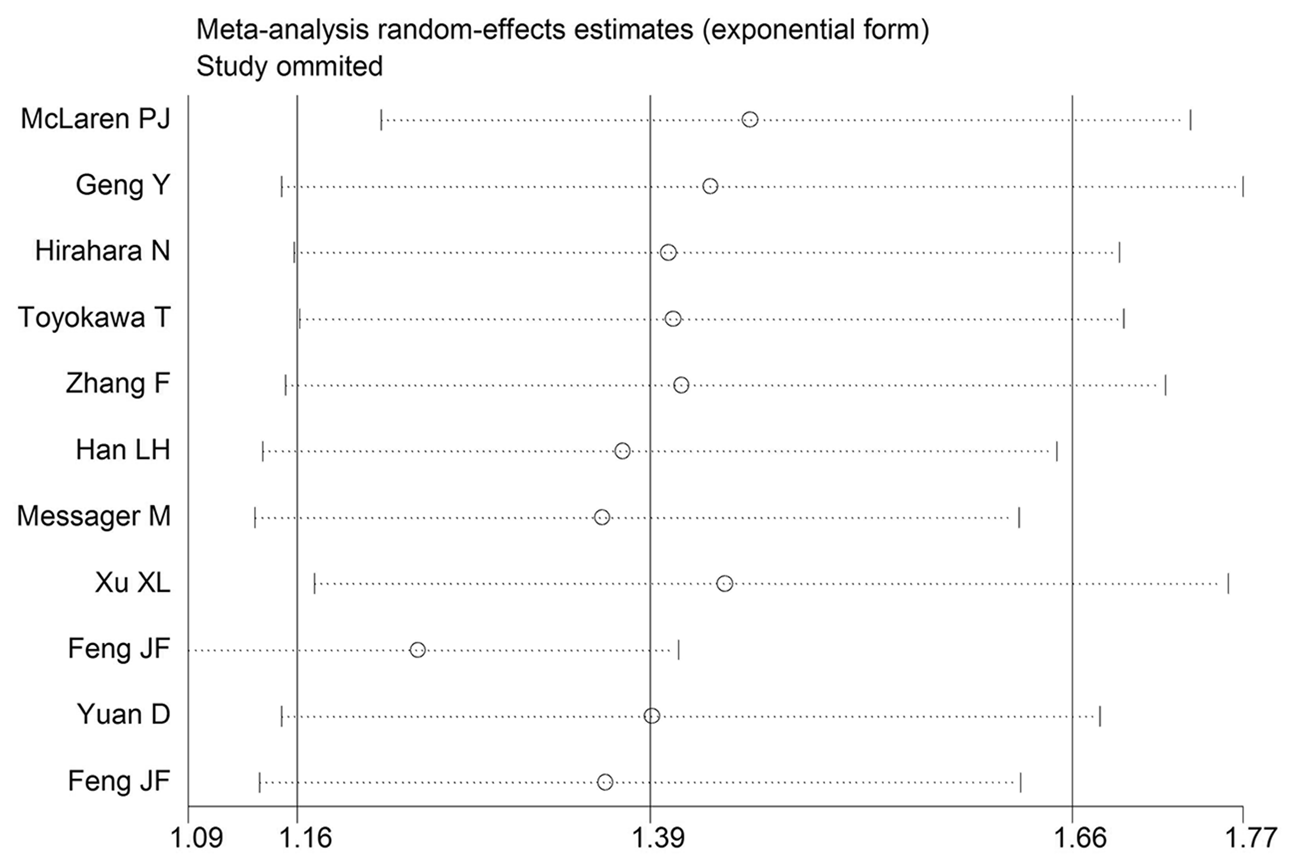
Sensitivity analysis for OS

Sensitivity analysis was performed by eliminating one study at a time and analyzing the remaining studies. The results are shown in Figure 3, the results were not substantially changed, showing the reliability and stability of our results. Furthermore, a meta-regression was also conducted to explore the potential factors that are responsible for heterogeneity in OS, The results showed that the above factors could partly explain the heterogeneity but did not reach statistical significance (Table 2).

### Publication bias

The Begg’s funnel plot and Egger’s linear regression test were performed to analyze the publication bias. No evidence of obvious publication bias existed in DFS/CSS (Pr>|z|=0.536 for Begg’s test and *P*>|t|=0.910 for Egger’s test). The P value of Egger’s test indicated that there was publication bias in OS (*P*>|t|=0.007) among these included studies. Therefore we further performed the “trim and fill” analysis. It was estimated that 1 study evaluating the prognostic value of PLR in OS remained unpublished. The filled meta-analysis concerning OS (HR=1.361, 95%CI: 1.143–1.621, p < 0.01).

## DISCUSSION

Our meta-analysis combined the outcomes of 6699 esophageal cancer patients from 16 studies (17 cohorts), indicating that elevated PLR significantly predicted poor OS (HR: 1.389, 95% CI: 1.161-1.663, *P*<0.001, Figure 2) of esophageal cancer patients.

Though with heterogeneity, subgroup analyses in our study showed that elevated PLR was an effective prognostic factor for poor OS of esophageal cancer patients who had received various types of treatment including surgical resection and mixed treatment. There is also a significant association between PLR and dichotomized cut-off value PLR≤160 or >160. In addition, our pooled results demonstrated that elevated PLR was associated with poor DFS (HR: 1.404, 95% CI: 1.169-1.687) and CSS (HR: 1.686, 95% CI: 1.146-2.480) in patients with esophageal cancer. Taking all of these in to consideration, PLR may be as a significant biomarker in the prognosis of esophageal cancer.

Tumor associated systemic inflammatory plays an important and multifaceted role in tumor prognosis [7, 9]. The exact mechanism between inflammation and tumor was still unknown [31]. Tumor-related inflammation causes suppression of antitumor immunity by recruiting regulatory T cells and activating chemokines, which results in tumor growth and metastasis. The presence of both thrombocytosis and neutrophilia tends to represent a nonspecific response to cancer-related inflammation [32]. Platelets can promote tumor progression by platelet-derived growth factor, platelet factor and thrombospondin [33, 34]. It has been suggested that interleukin-4 and -5 produced from tumor-infiltrating T cells in tumors, might promote tumor growth and spreading [4, 35]. Therefore, the relative value of a combined platelet and lymphocyte counts index (being expressed in the form of a PLR) can reflect the pro-tumor efficacy and antitumor capacity of the host more accurately [34, 36].

However, several disease conditions may affect PLR, including myocardial infarction, diabetes, renal diseases, inflammatory diseases, and infection, as well as some medications, such as antidiabetic, antibiotics drugs and cancer chemotherapy [37, 38]. The co-occurrence of these conditions may therefore affect the prognostic ability of PLR to predict survival outcomes.

There were several limitations in this meta-analysis. Firstly, the great majority of the enrolled studies were retrospective. Thus some biases, such as information bias, misclassification bias and selection bias, may be existed in the meta-analysis. Secondly, this meta-analysis was constrained to studies published in English. Strictly, some eligible studies published in other languages might be missed. In addition, Heterogeneity is a potential problem that may affect the interpretation of the results of all meta-analyses. The presence of heterogeneity may result from many other factors, including age distribution, ethnicity, gender, PLR cut-off value and so on. Theoretically, PLR could be affected by various pathological conditions, such as infection and medications, and varies from time to time.

In conclusion, our meta-analysis demonstrated that elevated PLR might be a poor prognostic factor for patients with esophageal cancer. Compared to other prognostic markers, PLR seems to be an inexpensive, widely-obtained, repeatable and reliable predictor for esophageal cancer patients. Esophageal cancer patients with high PLR may benefit from modifying inflammatory responses and modulating the immune system. In the future, more studies that are well-designed and large-scale are needed to confirm whether PLR has a prognostic value in patients with esophageal cancer.

## METHODS

### Search strategy

A comprehensive literature search was performed by using PubMed, Ovid, the Cochrane Library and Web of Science databases to evaluate the prognostic value of PLR in patients with esophageal cancer. Our search strategy included terms of: “PLR” (e.g., “platelet lymphocyte ratio”, “platelet to lymphocyte ratio”, “platelet-to-lymphocyte ratio”) and “esophageal neoplasm” (e.g., “esophageal cancer”, “esophageal carcinoma”, “esophageal squamous cell carcinoma”). The deadline of our primary search was September 30, 2017. In addition, the reference list was also scrutinized for further relevant articles.

### Inclusion and exclusion criteria

Inclusion criteria for the primary analysis was as follows: (1) patients with esophageal cancers in the studies were histopathologically confirmed; (2) the PLR was measured before treatment; (3) investigated the association of PLR with overall survival (OS), disease-free survival (DFS) or cancer-specific survival (CSS); (4) full text articles in English. Exclusion criteria was as follows: (1) abstracts, letters, editorials, case reports, reviews or nonhuman research; (2) the full text was not available in English; (3) studies with insufficient data for estimating HR and 95% CI; (4) studies had overlapping or duplicate data.

### Data extraction and quality assessment

All candidate articles were evaluated and extracted independently by two investigators (Zhao and Zhang). Any conflicts in data extraction or quality assessment were resolved by a third investigator. If the results reported had possible overlap, only the most recent or the most complete study was included in this study. We extracted data including: first author’s name, year of publication, country (region), sample size, gender, follow ups, cut-off value, treatment strategy, cancer type and HRs with 95% CIs. The quality of the included studies was assessed according to the Newcastle-Ottawa Quality Assessment Scale [39], which includes three aspects of evaluation: selection (0-4 points), comparability (0-2 points), and outcome assessment (0-3 points). NOS scores of ≥6 were assigned as high-quality studies.

### Statistical analysis

HR and 95%CI were obtained directly from each literature or estimation according to the methods by Parmer et al [40]. A HR>1 indicated a worse prognosis in esophageal cancer patients with high expression of PLR. For each meta-analysis, Cochran’s Q test and Higgins I-squared statistic were undertaken to assess the heterogeneity of the included trials. *I*^2^>50% is considered as a measure of severe heterogeneity. Both fixed-effects (Mantel–Haenszel method) and random effects (DerSimonian–Laird method) models were used to calculate the pooled HRs and 95%CIs. The random-effects model was used if there was heterogeneity between literatures; otherwise, the fixed-effects model was adopted.

Subgroup analysis was conducted to explore and explain the diversity (heterogeneity) among the results of different studies. Publication bias was assessed by Begg’s funnel plot and Egger’s bias test [41]. All P values were two-tailed, A *P*<0.05 was considered statistical significant. Statistical analyses were performed using STATA statistical software package version 12.0 (STATA, College Station, TX, USA).
